# Towards robust cell culture processes — Unraveling the impact of media preparation by spectroscopic online monitoring

**DOI:** 10.1002/elsc.201900050

**Published:** 2019-08-22

**Authors:** Matthias Brunner, Philipp Brosig, Monika Losing, Marco Kunzelmann, Amandine Calvet, Fabian Stiefel, Jan Bechmann, Andreas Unsoeld, Jochen Schaub

**Affiliations:** ^1^ Bioprocess Development Biologicals Boehringer Ingelheim Pharma GmbH & Co. KG Biberach Germany; ^2^ Analytical Development Biologicals Boehringer Ingelheim Pharma GmbH & Co. KG Biberach Germany

**Keywords:** cell culture, media preparation, media quality, PAT, process robustness, scale‐up

## Abstract

Biopharmaceutical manufacturing processes can be affected by variability in cell culture media, e.g. caused by raw material impurities. Although efforts have been made in industry and academia to characterize cell culture media and raw materials with advanced analytics, the process of industrial cell culture media preparation itself has not been reported so far. Within this publication, we first compare mid‐infrared and two‐dimensional fluorescence spectroscopy with respect to their suitability as online monitoring tools during cell culture media preparation, followed by a thorough assessment of the impact of preparation parameters on media quality. Through the application of spectroscopic methods, we can show that media variability and its corresponding root cause can be detected online during the preparation process. This methodology is a powerful tool to avoid batch failure and is a valuable technology for media troubleshooting activities. Moreover, in a design of experiments approach, including additional liquid chromatography–mass spectrometry analytics, it is shown that variable preparation parameters such as temperature, power input and preparation time can have a strong impact on the physico‐chemical composition of the media. The effect on cell culture process performance and product quality in subsequent fed‐batch processes was also investigated. The presented results reveal the need for online spectroscopic methods during the preparation process and show that media variability can already be introduced by variation in media preparation parameters, with a potential impact on scale‐up to a commercial manufacturing process.

**Abbreviations**
2D FL: 2D fluorescence
DO: dissolved oxygen
DoE: design of experiment
EEM: excitation‐emission matrix
F‐media: feed media
MIR: mid‐infrared
ORP: oxidation reduction potential
P‐media: production/basal media
P/V: specific power input

## 1 INTRODUCTION

Robust biopharmaceutical processes are a prerequisite in order to achieve a consistent volumetric productivity and product quality. However, cell culture processes can be affected by different sources of variation. Besides analytical and biological variability, a major source for variable process performances is often reported to be linked to the chemical media composition 1, 2, 3. Cell culture media often consist of more than 50 components, including substances with known stability issues such as cysteine, insulin, and riboflavin 4, 5, 6. Quality control for cell culture media may include raw material screening with spectroscopic methods as near‐infrared and mid‐infrared (MIR) spectroscopy 7, 8. However, the final liquid media composition is commonly only assessed by pH and osmolality measurements as well as limited single nutrient analytics (e.g. glucose measurement). These methods do not represent the complex nature of liquid cell culture media and appear insufficient for reliable evaluation of the media quality. Spectroscopic methods such as offline 2D fluorescence (2D FL) spectroscopy have been described in various publications to have the potential for rapid quality assessment of liquid cell culture media 5, 9, 10, 11, 12, however are not routinely used in industry. These offline analytics are furthermore time consuming and labor intensive and might be affected by sample handling as well as freeze/thawing procedures. In the first study of this contribution, we compare MIR and 2D FL spectroscopy as online monitoring tools in cell culture media preparation for industrial manufacturing of biologics. The application of online spectroscopy during the preparation process can indicate media quality online and at the same time provide increased understanding of the preparation process which can finally indicate potential root causes for media variability. Media troubleshooting activities are usually time consuming, using limited data sets, and are often also inconclusive and therefore would benefit strongly from spectroscopic online monitoring methods.

**PRACTICAL APPLICATION**

Media variability can strongly affect biopharmaceutical production processes. In this publication we show that 2D fluorescence spectroscopy is a powerful tool to detect media variability and as an online application can strongly support media troubleshooting activities. Furthermore, this methodology can act as a media batch release method and can be used for characterization of media preparation processes for transfer or scale‐up/down activities. Data analysis further revealed strong correlations of the 2D fluoresence signals with several anayltes of the LC‐MS method. Therefore, 2D fluorescence could be even used to predict changes in media composition based on the PLS regression models. Moreover, the presented data clearly demonstrates that media variability can be introduced during the media preparation process by application of variable preparation parameters. In summary, the results clearly highlight the need for more advanced and online media characterization methods as well as defined media preparation procedures and scale‐up strategies.

Besides raw materials, another source for media variability might be derived from the application of different media preparation parameters (e.g. specific power input [P/V], temperature, and preparation time) at different scales, facilities, and types of preparation vessels. Scale‐dependent effects such as different surface to volume ratios can affect the powder–liquid interaction/dispersion and therefore can have a strong impact on the overall preparation process. Moreover, preparation parameters such as preparation time, temperature, and P/V can not be kept constant between all scales and preparation vessels but can strongly affect gas solubility/entry, powder dispersion, and chemical reaction kinetics 13, 14. Although there is literature on the chemical degradation of cell culture media or impact of aged media on process performance 2, 5, 6 available, to our knowledge there is no reported study investigating the effect of media preparation parameters on media quality. Therefore, the second study in this contribution focuses on the impact of variable media preparation parameters on cell culture media and subsequent fed‐batch processes using a design of experiments (DoE) approach.

Finally, our results demonstrate that spectroscopic online methods during the media preparation process can significantly increase process robustness and improve the understanding of the media preparation process. Furthermore, we could demonstrate that media variability might be already introduced during the media preparation process by application of different preparation parameters.

## 2 MATERIALS AND METHODS

### 2.1 Media preparation procedure

Cell culture media preparations were performed in a modified 3 L stirred tank bioreactor system (Mobius^®^, Merck Millipore, Germany). Modifications, in order to mimic large‐scale media preparation, included adjustment of the head plate, a stainless steel inlet, and stainless steel stirrer shaft. Production/basal media (P‐media) and feed media (F‐media) were prepared according to the specific media recipe and with controlled temperature, P/V and under light protected conditions. Sterile filtration of the media were conducted with the same filter cartridges and lots (FF‐Millipak 60, 0.1 µm, Merck Chemicals, Germany) for all media batches. Osmolality measurements were performed after sterile filtration (Osmomat^®^ 030, Gonotec GmbH, Germany) and media were finally stored at 4°C under light protected conditions. Within each study, if not explicitly stated differently, the same lots of media components were used in order to prevent raw material lot‐to‐lot variability.

### 2.2 Analytical methods for media characterization

Media preparation processes of the first study were monitored with a pH‐probe (EasyFerm^®^, Hamilton, USA) and two different spectroscopic monitoring methods. Fluorescence was measured with a 2D FL probe (BioView^®^, DELTA, Denmark) with an excitation range from 290 to 550 nm and an emission range from 310 to 590 nm. The measurement increment for the corresponding excitation‐emission matrix (EEM) was set to 20 nm with a gain of 1500 and a sampling time of 1 min per EEM. MIR measurements were conducted by a ReactIR™ iC10 probe (Mettler Toledo, Switzerland) with a silver halide glass fiber. The FTIR instrument consisted of an ATR diamond tipped probe. The measurement range was set between 650 and 2000 cm^−1^ at a resolution of 5 cm^−1^. The sampling time was 1 min per measurement. The used mercury cadmium telluride (MCT) detector was cooled with liquid nitrogen. Data analysis was only performed in the fingerprinting region between 800 and 1500 cm^−1^.

The media preparations of the second study were monitored by a pH and 2D FL probe as described above and additionally with an oxidation reduction potential (ORP) probe (EasyFerm^®^ Plus ORP Arc 120, Hamilton, USA) and a dissolved oxygen (DO) probe (VisiFerm^®^ DO 225, Hamilton, USA). The pCO_2_ was measured offline at the end of the media preparation with a blood gas analyzer (RAPIDLab^®^ 348EX, Siemens, Germany).

### 2.3 Data evaluation and DoE

Data preprocessing of the MIR data was performed in SIMCA^®^ (Sartorius Stedim Biotech, Germany) by background subtraction, standard normal variate, Savitzky‐Golay, and 1^st^ derivative for the principal component analysis (PCA) models. MIR online time‐series of the partial least square (PLS) models was preprocessed with background subtraction, Savitzky‐Golay, and 2^nd^ derivative. The preprocessing methods are described in more detail in refs. 15, 16. PLS models from MIR online data were further processed by exclusion of wavelengths with a Variable Importance in Projection value below 0.5. Data preprocessing of the 2D FL data was done by an in‐house code in order to remove scattering effects (e.g. Rayleigh scattering) and for background subtraction 17. All PCA and PLS models were established using SIMCA^®^ (Sartorius Stedim Biotech, Germany). The goodness of fit (*R* ^2^) and goodness of prediction (*Q* ^2^) are presented for each model. PLS regression models, of the 2D FL excitation‐emmision‐matrix with the LC‐MS data, additionally include information about the root mean square error of estimation (RMSEE) and root mean square error from cross‐validation (RMSEcv).

Cell specific rates were calculated analogous to previously published articles 18. The DoE was established using the software Design‐Expert^®^ (v10) (Stat‐Ease, USA). The P‐media DoE was based on a D‐optimal screening design with the factors P/V, temperature, and preparation time. The factor preparation time was controlled in a way that each addition step during the preparation was prolonged according to the percentage increase in total preparation time compared to the minimum preparation time. The minimum preparation time was determined at the lowest temperature and P/V settings with an focused beam reflectance measurement (FBRM) particle probe (Mettler Toledo, Germany) to ensure proper dissolution of each media compound before the addition of the next one. Due to the known impact of media age on media composition and process performance 2, 6, the factor storage time was included in the DoE as a covariate. The covariate vector was designed to be as orthogonal as possible to the existing design to reduce possible correlations to other factors. The F‐media DoE was based on an I‐optimal optimization design with the same factors as the P‐media DoE. Within each DoE, the same lots of media components were used in order to avoid raw material lot‐to‐lot variability. The specific preparation conditions for each media batch are presented in Table 1. The results of the DoE are presented as cofficients in terms of coded factors. These coefficients are useful for identifying the relative impact of the factors and can be used to make predictions about the response for given levels of each factor, whereby the high levels of the factors are coded as +1 and low levels as −1.

**Table 1:** Conditions of the P‐media and F‐media design of experiments. The P‐media experiments are based on a D‐optimal DoE with several replicates along the covariate factor storage time. The F‐media experiments are based on an I‐optimal DoE with less replicates but additional levels for each factor

P‐media No.	N1	N2	N3	N4	N5	N6	N7	N8	N9	N 10	N 11	N 12	N 13	N 14	N 15	N 16	N 17	N 18
Temperature [°C]	38	28	38	28	38	28	28	38	28	28	38	28	38	38	28	28	38	28
Preparation time [min]	160	65	65	160	65	160	65	160	65	65	160	160	160	65	160	65	65	65
P/V [W/m^3^]	130	130	30	30	130	130	30	30	30	130	30	130	130	130	30	130	30	130
Storage time [d]	18	18	17	17	14	14	12	12	11	11	10	7	6	6	5	5	4	4

F‐media No.	N1	N2	N3	N4	N5	N6	N7	N8	N9	N10	N11	N12	N13	N14	N15	N16	N17	N18
Temperature [°C]	37	30	33.5	30	33.5	37	33.5	33.5	30	37	37	33.5	37	33.5	30	37	33.5	30
Preparation time [min]	110	140	140	170	170	170	110	140	110	140	170	170	140	140	110	110	140	170
P/V [W/m^3^]	110	88	64	110	88	64	88	64	64	88	110	88	88	110	110	64	88	64
Storage time [d]	14	19	19	17	10	18	17	12	13	18	13	14	7	12	11	10	7	11

P/V, specific power input.

### 2.4 LC‐MS analytics of vitamins and reaction products

Vitamins and several reaction products in P‐media samples were measured with a LC‐MS/MS method. A complete list of the reported components is presented in the Supporting Information Table 1. P‐media samples of the DoE study (Table 1) were frozen directly after media preparation and stored at −70˚C. Samples were thawed at room temperature and under constant protection from light exposure. All samples were measured in triplicates using an InfinityLab Poroshell Hydrophilic interaction chromatography‐Z column (Agilent Technologies, USA). MS/MS was performed on a QTRAP^®^ 6500+ triple quadrupole mass spectrometer (Sciex, USA) equipped with an ESI source and coupled to an Agilent 1260 series binary HPLC system (Agilent Technologies, USA). Nitrogen was used as curtain and collision gas. Data was acquired using MultiQuant 3.0.3 software (Sciex, USA).

### 2.5 Seed train and fed‐batch cultivations

Two CHO (chinese hamster ovary) cell lines (Cell line A and B) producing a monoclonal antibody (mAb) were cultivated in the P‐ and F‐media batches. Seed train cultures were processed in shake flasks. Fed‐batch cultivations were conducted for 13 or 14 days in an ambr^®^15 (Sartorius Stedim Biotech, Germany) bioreactor system. The same F‐media batch was used in the P‐media DoE cultivations and the same P‐media batch in the F‐media DoE cultivations. Feed media was added continuously from day 1 until the end of the process. P‐media was equilibrated at process conditions for 24 h prior inoculation. Glucose was added to the processes on demand. Cultivation samples were taken every 24 h and cell counting and viability determination was performed using a Cedex HiRes analyzer (Roche, Germany). Metabolites glucose, lactate, and ammonia were determined with a Konelab™ Prime60i (Thermo Scientific, USA) or Biosen S‐line (EKF‐diagnostics GmbH, UK) device. Antibody concentration was determined with a Protein‐A HPLC method (Thermo Scientific, USA) beginning at day 8 or 10 of the processes. Antibody charge variants were analyzed using a ProPac WCX‐10 (4 × 250 mm) analytical column (Thermo Fischer Scientific, USA) connected with a HPLC system (Waters, USA) with UV detection at 280 nm. SEC was performed with a BEH200 SEC column (Waters, USA) connected to a HPLC System (Waters, USA) with UV detection at 280 nm. N‐glycan determination was performed using a LabChip GXII (PerkinElmer, USA) and HT Glycan Reagent Kit (PerkinElmer, USA).

## 3 RESULTS AND DISCUSSION

### 3.1 Spectroscopic online monitoring of cell culture media preparation–1^st^ study

The goal of the first study was to evaluate two spectroscopic online monitoring methods with respect to their suitability in cell culture media preparation. One major criterion was if the spectroscopic methods can detect differences in media batches online during the preparation process. MIR and fluorescence spectroscopy were selected due to their high sensitivity 19, 20, 21. In total, nine media batches were prepared and variability in the media preparation was induced through usage of basal powder from different suppliers (SS1, SS2), different production batches of media component 4 (LOT), as well as differences in trace element concentrations (TE) and variable preparation temperature (LT, HT) (Table 2).

**Table 2:** Variability introduced during P‐media preparation in order to evaluate different spectroscopic methods as online monitoring tools. Temperature settings were kept constant throughout the media preparation process. All media recipes and sources were identical between media batches despite the intended introduced variability described in this table

Abbreviation	Experiment name/media variability	Temperature [°C]
REF 1 ‐ 3	Reference media preparation (3x)	33
SS1	Second supplier 1 basal powder	33
SS2	Second supplier 2 basal powder	33
LOT	Media component 4 of a different lot	33
TE	Media component 4 surrogate with a different trace element concentration	33
LT	Low temperature	25
HT	High temperature	38

#### 3.1.1 Fingerprint at the end of the media batches

**Figure 1:**
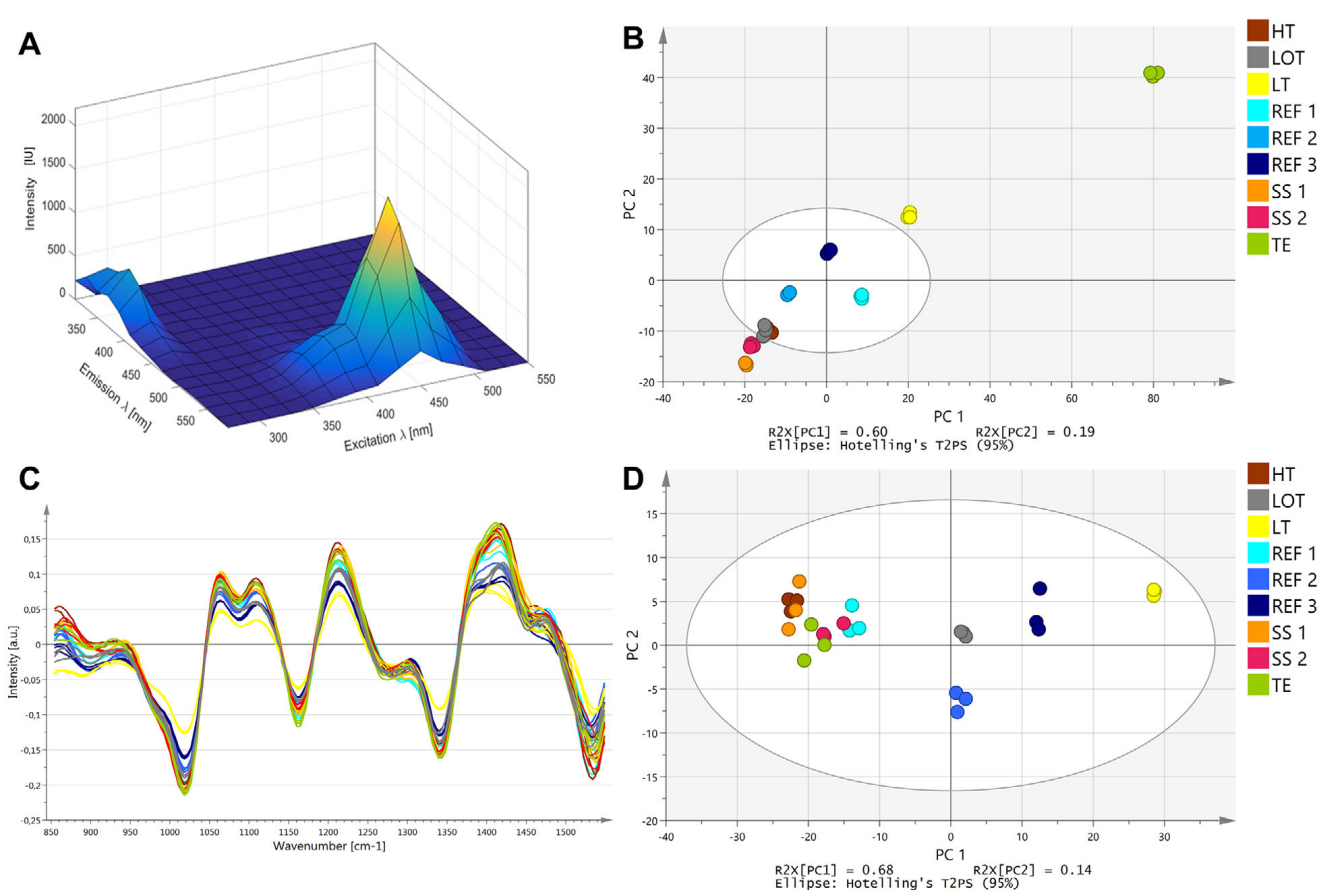
(A) An example of a 2D FL EEM of cell culture P‐media at the end of the preparation process. (B) PCA model with principal component 1 (PC 1) and principal component 2 (PC 2) (*R* ^2^:0.78; *Q* ^2^:0.62) derived from the 2D FL data at the end of the preparation process. The model is based on the reference P‐media batches, media batches that are outside of the Hoteling's *T* ^2^ of the model are considered significantly different from the reference media. (C) Overlay of MIR spectra of all P‐media batches at the end of the preparation process. Spectra were preprocessed via standard normal variate (SNV), Savitzky‐Golay (SG), and 1^st^ derivative. (D) PCA model with principal component 1 (PC 1) and principal component 2 (PC 2) (*R* ^2^:0.86; *Q* ^2^:0.68) derived from the MIR data at the end of the preparation process. The model is based on the reference P‐media batches. All media batches are inside the Hoteling's *T* ^2^ of the model and are therefore not significantly different form the reference media. A detailed description of the figure legend is presented in Table 2. *Q* ^2^, goodness of prediction; *R* ^2^, goodness of fit

An example of a 2D FL EEM at the end of a media batch is given in Figure 1A for the media batch TE. The final EEM matrix of the TE batch as well as all other media batches is mainly characterized by two peaks with excitation/emission maxima around ex450 nm/em530 nm and ex290 nm/em370 nm. Based on the EEM data of the three reference media batches (REF 1–3), a PCA model was established and the other media batches were compared relative to this model space (Figure 1B). The media batches SS 1, SS 2, TE, and LT were located outside of the Hoteling's T^2^ of the model and were therefore significantly different from the reference media. Based on the PCA model, the basal powder of the second supplier, included in media batches SS 1 and SS 2, was investigated in more detail. The 2D EEM matrix of the second supplier media batches had a decreased peak at ex450 nm/em530 nm when compared to the reference media (data not shown). This peak is known to be strongly influenced by riboflavin 5. Finally, the riboflavin content of the reference media basal powder and the second supplier basal powder were analyzed and the results confirmed that the riboflavin content in the reference media was indeed significantly elevated in comparison to the second supplier basal powder. The fingerprint of the TE media batch was clearly outside of the Hoteling's T^2^ of the reference media preparations (Figure 1B). The main difference between these batches was based on the peak at ex290 nm/em370 nm which can be correlated to the amino acids tryptophan (max. ex280, 290 nm/em350 nm), tyrosine (max. ex275, 280 nm/em300, 330–350 nm), and phenylalanine (max. ex260 nm/em280 nm) 22, 23, 24. The strong difference in the 2D FL data most probably derived from the different trace element concentrations of the media batches. TE media had a reduced iron content which can have a strong effect on tryptophan fluorescence signals via quenching 25. The fact that the media batch LT was significantly different from the reference media batches can be explained by the temperature dependence of the fluorescence signals 26 or might derive from different reaction kinetics at lower temperature.

Figure 1C presents the preprocessed MIR signals of all media batches at the end of the preparation process. Similar to the 2D FL signals, the last three measurements of each batch were used for Figure 1C and the corresponding PCA models in Figure 1D. All media batches are inside the Hotellings T^2^ of the model and are therefore not different from the reference media preparations. The fact that none of the induced media variability parameters led to a change in the final MIR signal when compared to the reference media can be due to the fact that the induced variability was not strong enough to create changes in the MIR signal. Although MIR is generally a sensitive method for detection of a wide range of analytes, it has limitations as it is insensitive to trace element variations and very low concentration‐dependent differences. Other studies have shown that glucose concentrations can be predicted via MIR in cell cultivations with a standard error of prediction of 0.27 21 and 0.16 g/L 27. Furthermore, MIR measurements can be impacted by baseline offsets, drifts, and scattering effects 20 and need to be preprocessed. However, some of the useful spectroscopic information might be removed by preprocessing from the MIR signals resulting in no detectable differences between media batches.

Based on the 2D FL data at the end of the media preparation, it can be concluded that some media batches were different from the reference media. In a scenario in which the variability was not introduced on purpose, this would mean that differences between batches can be detected, however without knowledge of the underlying root cause. Yet, with the help of the spectroscopic online data further insight into the preparation process can be obtained.

#### 3.1.2 Root cause analysis via spectroscopic online data

**Figure 2:**
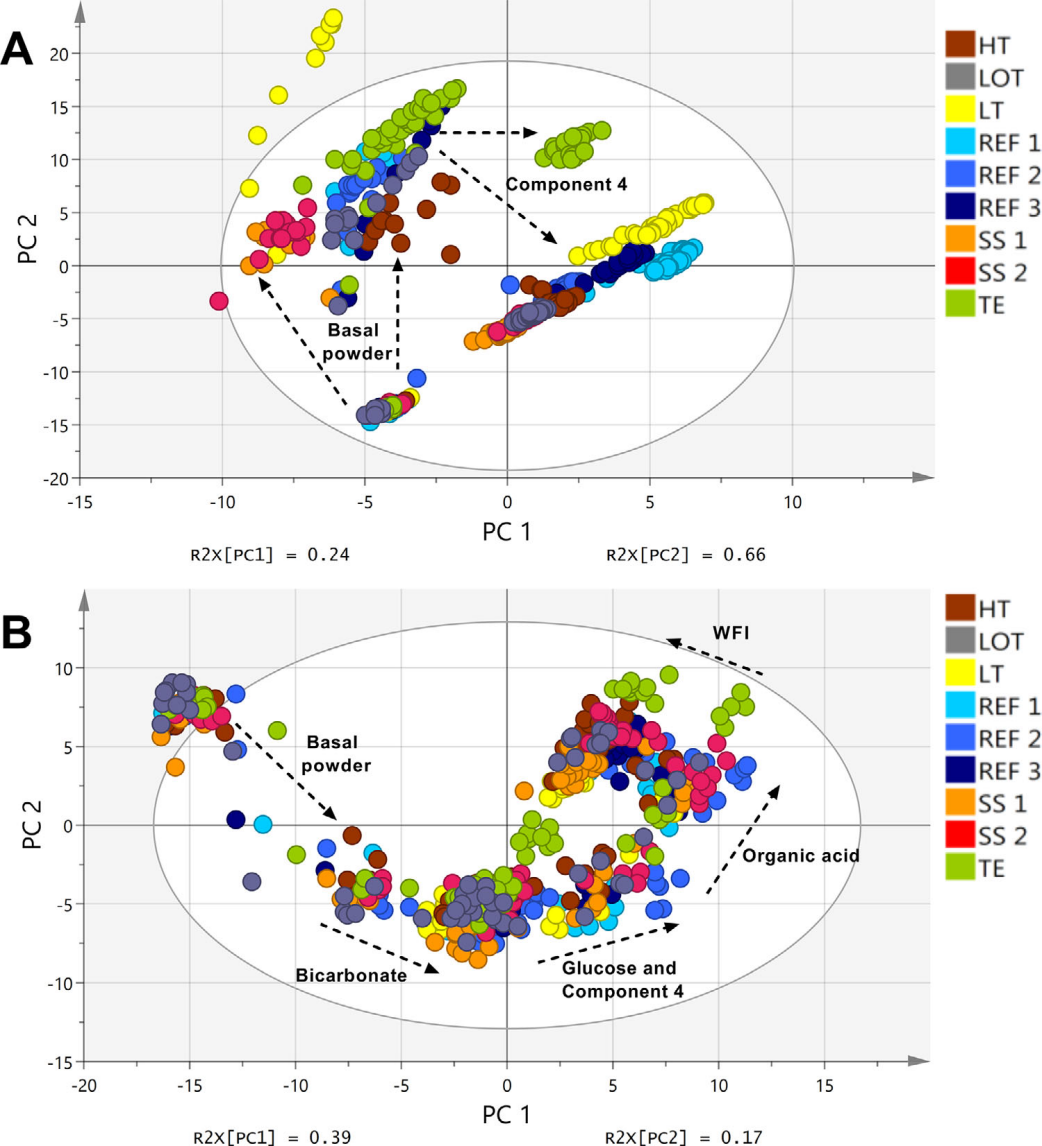
(A) PLS model with principal component 1 (PC 1) and principal component 2 (PC 2) (*R* ^2^X: 0.90; *R* ^2^Y:0.47; *Q* ^2^: 0.27) derived from the 2D FL data over time of all P‐media batches. The addition of basal powder and Component 4 led to strong changes in the 2D FL signal during the preparation. (B) PLS model with principal component 1 (PC 1) and principal component 2 (PC 2) (*R* ^2^X: 0.56; *R* ^2^Y:0.83; *Q* ^2^: 0.72) derived from the MIR data over time of all P‐media batches. Data were preprocessed via Savitzky‐Golay (SG) and 2^nd^ derivative. Changes in the MIR profile could be detected mainly after addition of basal powder, bicarbonate, glucose, Component 4, organic acid, and water for injection (WFI). A detailed description of the Figure legend is presented in Table 2. *Q* ^2^, goodness of prediction; *R* ^2^, goodness of fit

Figure 2A presents the online data of the 2D FL signal comprised in a PLS model for all media batches. Each score on the scores‐plot represents one measurement during the preparation process. At the beginning of the media preparation process all signals are close together. This is expected since the preparation processes start with pure water and therefore no fluorescence signals occurred. After the addition of the basal powder, however, differences between the reference media batches (REF 1, 2, 3) and media batches SS1, SS2, as well as LT could be detected. The separate cluster of media preparations SS1 and SS2 after basal powder addition (Figure 2A) reinforces the fact that the final differences between the media batches at the end of the preparation, as observed in Figure 1B, derive from the different basal powder. The fluorescence signals of the media batch LT were different from the other media batches directly after basal powder addition. Since the same basal powder was used for this media batch as for the reference media batches, the difference occurred most probably only due to the different temperature set points during the preparation (Table 2). The fluorescence signals of media batch TE, which was significantly different from the reference media at the end of the preparation (Figure 1B), were comparable to the reference media after basal powder addition. However, after the addition of component 4, the fluorescence signal of the TE batch strongly deviated from the reference media preparations (Figure 2A). Therefore, the differences between the media batches TE and REF 1, 2, 3 at the end of the media batches could be directly linked to the differences in component 4. As stated earlier, reduced iron concentrations in component 4 in media batch TE most probably led to different quenching effects on the tryptophan fluorescence signal 25. In general, the fluorescence signal during the media preparation processes changed mainly after addition of the basal powder and component 4 for all media batches.

The online MIR data for all media batches is presented in Figure 2B. Similar to the fluorescence data, each score on the scores‐plot represents one measurement during the entire media preparation. In general, the MIR signals showed strong changes in their signals after addition of basal powder, bicarbonate, glucose, component 4, organic acid, and water for injection (WFI) (Figure 2B). Due to the fact that the MIR method is based on vibrational signals of general chemical bonds, e.g. C‐C bonds, the method can detect various different chemicals, whereas 2D FL measurements are limited to fluorescence signals. Although no differences at the end of the media batches could be detected based on Figure 1D, one difference could be observed in the online PLS‐model. The MIR signal of the media batch TE was different from the other media batches after addition of component 4. Due to the fact that the MIR method is not sensitive to trace elements, the different MIR signals most probably derived from the fact that a surrogate mixture with reduced complexing agents was used in comparison to the other media batches. The other media batches, however, showed comparable signals in the online MIR data.

#### 3.1.3 MIR and 2D fluoresence comparison

In comparison to the MIR method, the 2D FL sensors have the advantage that data preprocessing is much simpler and therefore the final PCA or PLS models are far less dependent on the specific preprocessing method that was used to build the models. Furthermore, the induced variability in our experiments could only be resolved with the 2D FL method. However, this is strongly dependent on the induced variability since both methods rely on different measurement principles and can therefore deviate in their sensitivity in respect to different media components. A comparison of both methods based on the presented results and literature is shown in Table 3. Theoretically, the application of multiple spectroscopic methods and data‐fusion models as used in other studies 28 would be best to detect differences between media batches, however this approach is impractical for large‐scale media production processes.

**Table 3:** Comparison of MIR and 2D FL online spectroscopy for application in cell culture media preparation based on the presented results and literature 19, 20, 56

	MIR	2D FL
Sensitivity	High	High–very high
Spectral information	General chemical properties	Fluorescence signals only
Data preprocessing	Complicated, may mask effects	Simple in comparison to MIR
Further advantages/disadvantages	Water potentially masks effects; fragile and expensive equipment	Limited supplier for 2D FL probes available
Capability for detection of media variability (this study)	Low–medium	High

2D FL, 2D fluorescence; MIR, mid‐infrared.

### 3.2 Impact of preparation parameters on media quality–2^nd^ study

Reproducible and scalable media preparation is a challenging task 4. Media preparation processes can vary in their specific preparation parameters dependent from scale and type of preparation vessel. This variability during the preparation process might lead to media variability and consequently process variability. The goal of the second study was therefore to investigate the effect of scale‐up critical preparation parameters such as temperature, specific power input, and preparation time. These parameters might not be constant between all scales and facilities, especially the preparation time in large‐scale is much longer than in small‐scale due to different mixing characteristics and surface to volume ratios of the preparation vessels. The impact of preparation parameters on media quality was investigated in a comprehensive DoE approach. Due to the fact that not all media batches could be prepared at the same time and since media storage is known to have an effect on media characteristics 2, 6, 29, the factor storage time was included as a covariate in the DoE.

#### 3.2.1 Online monitoring of pH, ORP, and DO

**Figure 3:**
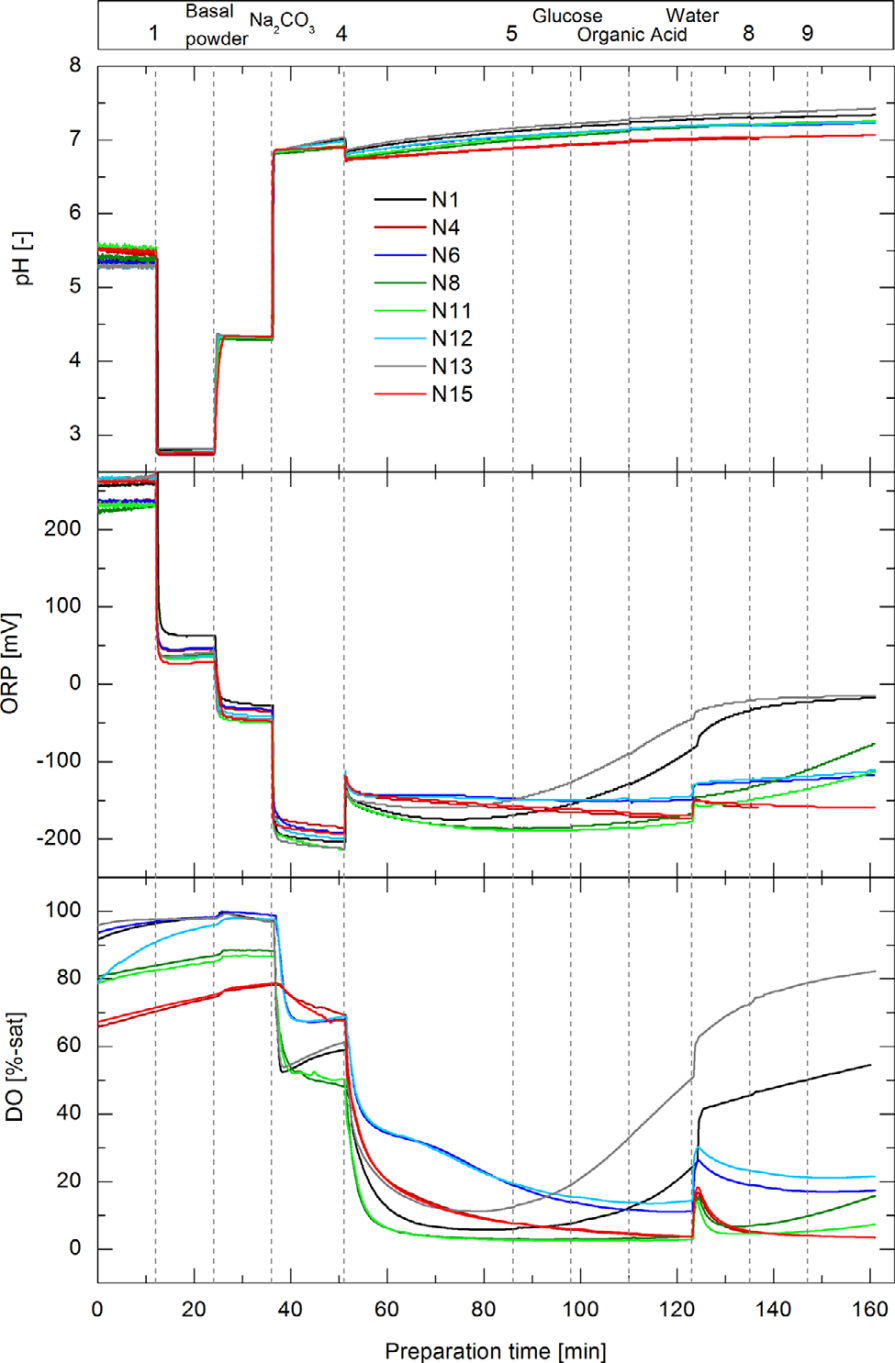
DO, ORP, and pH‐signal over preparation time for all P‐media preparations with 160 min of preparation time. Additional steps of media components are indicated by the vertical dashed lines and corresponding components are shown in the upper bar by their names or coded numbers. The legend corresponds to the media batch number out of Table 1

During the preparation process several physico‐chemical parameters were measured online as DO, ORP, and pH. Figure 3 presents the pH, ORP, and DO signals over preparation time for the P‐media preparations with 160 min of preparation time. The DO and ORP values at the end of the preparation processes varied strongly between the specific preparation conditions with ORP values between −15 and −190 mV. Since ORP values are strongly dependent on DO and pH 30, the strong increase in the ORP signal at the end of media batches N1 and N13 were most probably strongly influenced by the increase in the DO signals. The increase in DO during these media batches might be due to a saturation of oxidation reactions occurring at high temperature, high power input, and long preparation times. The DO and ORP signals decreased immediately after the addition of component 4, which contains trace elements (e.g. iron). This indicates that rapid oxidation reactions occurred which were triggered by the addition of trace elements 31. Several oxidation reactions can occur in cell culture media including the oxidation of cysteine to cystine 30, pyruvate oxidation 32, methionine oxidation 33, and tyrosine/tryptophan oxidation in the presence of riboflavin 34. Ascorbate, flavonoids, other polyphenolic components, and thiols can readily participate in oxidation reactions and form reactive oxygen species 31. The addition of component 4 led to strong changes in the ORP and DO signal as well as in the MIR and 2D FL signals presented in Figure 2, this further indicates that strong chemical changes occured in the media upon addition of this component. The pH values of the media batches started to deviate from each other after the addition of bicarbonate (Figure 3). Therefore, the pH variability was suspected to be correlated with different pCO_2_ stripping behavior at different preparation parameters. Gas exchange/transfer and gas solubilities are dependent on temperature and power input and therefore directly influence DO and pCO_2_ concentrations 35. This further explains the different DO values at the beginning of the media batches.

**Figure 4:**
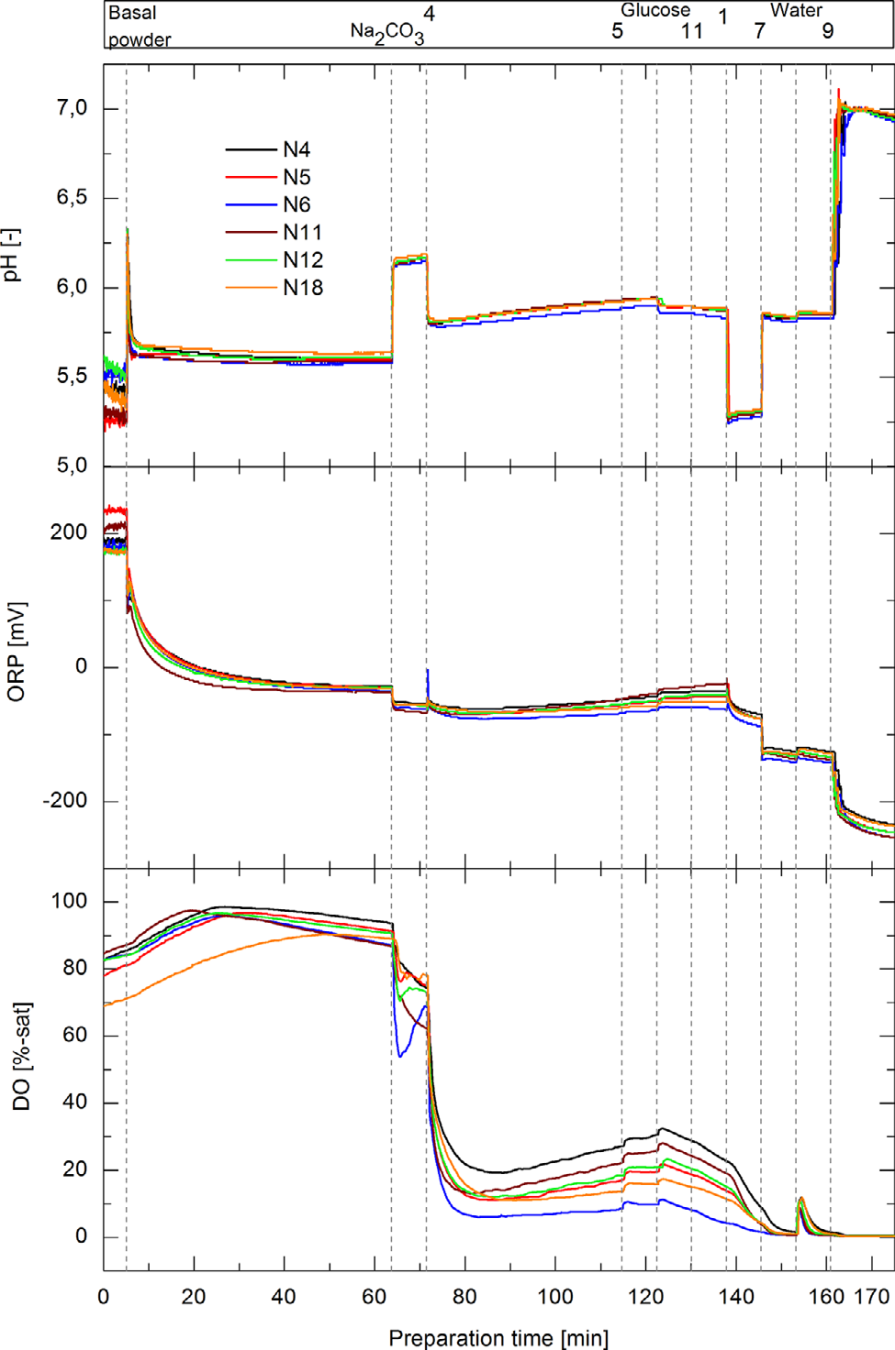
DO, ORP, and pH‐signal over preparation time for all F‐media preparations with 170 min of preparation time. Additional steps of media components are indicated by the vertical dashed lines and corresponding components are shown in the upper bar by their names or coded numbers. The legend corresponds to the media batch number out of Table 1

Figure 4 presents the pH, ORP, and DO signals over preparation time for the F‐media preparations with 170 min of preparation time. Similar to the P‐media preparation, the DO value started to decrease strongly after the addition of component 4. The ORP value however did not show a dramatic change in its signal. Interestingly, the DO, pH, and ORP values of all media batches were all similar at the end of the media batches. Although the DO value and further the ORP value started to deviate from each other after the addition of component 4, all values became similar after around 150 min of preparation time. The difference in the profiles between P‐ and F‐media can be attributed to the different media components, order of addition steps, component concentrations, and range of the DoE factors. The DO signal of all F‐media batches increased or plateaued, after the instant decrease due to addition of component 4, whereas the DO of the P‐media batches mostly further decreased over time after addition of component 4. Due to a higher concentration of component 4 in the F‐media compared to the P‐media, reaction kinetics for all potential oxidation reactions could have been elevated. Hence, reaction equilibrium could have been reached during the observed time in the F‐media, independent from the investigated preparation parameters. Regarding the DO, ORP, and pH profiles, all F‐media batches seemed to be similar at the end of the preparation and therefore more robust in response to variable preparation parameters than the P‐media batches.

#### 3.2.2 Impact on physico‐chemical media characteristics including LC‐MS and 2D FL data

A summary of the coded factors out of the DoE analysis for P‐media is presented in Table 4. The data table includes physico‐chemical measurements that were performed at the end of the preparation processes, except the DO average response which is based on the average DO value during the preparation process.

**Table 4:** Impact of preparation parameters on physico‐chemical media characteristics (P‐media). The coefficients in terms of coded factors of the DoE are presented for P‐media. Confidence interval was set to 95% for all responses. Values in italic are not significant. Interaction effects are denoted as A × C, A × B, and B × C

		(A)	(B)	(C)					
P‐media	Range	Temp. (28–38°C)	P/V (30–130 W/m^3^)	Prep. time (65–160 min.)	A × C	A × B	B × C	*R*²	*Q*²
Osmolality	276–288 [mOsm/kg]	−2.08	−2.03	−2.83	–	–	–	0.71	0.50
pH	7.0–7.37 [‐]	0.07	0.07	0.08	–	–	–	0.82	0.69
pCO_2_	7–32 [%]	−4.26	−3.30	−4.78	–	1.00	0.58	0.99	0.98
ORP	−15–−190 [mV]	17.94	25.10	28.40	22.85	7.63	6.68	0.98	0.94
DO _average_	27–52 [%]	−2.7	6.62	–	–	–	–	0.84	0.77
DO	2–82 [%]	*4.98*	13.03	6.48	9.20	–	*5.17*	0.79	0.49
2D‐FL (ex450/em530)	1700–2100 [IU]	−76.31	−24.94	−22.56	−33.27	–	–	0.86	0.71
2D‐FL (ex290/em370)	116–160 [IU]	−3.02	–	–	–	–	–	0.91	0.90
l‐methionine sulfoxide	44–100 [%]	−13.63	5.04	*0.39*	–	–	5.32	0.93	0.84
5‐hydroxyl‐l‐tryptophan	67–100 [%]	−6.59	−3.75	−3.88	–	–	–	0.76	0.57
Pyridoxal	64–100 [%]	−5.40	–	−5.28	–	–	–	0.64	0.45
Pyridoxamine	64–100 [%]	7.61	−*3.11*	5.06	3.72	–	–	0.79	0.53

DO, dissolved oxygen; ORP, oxidation reduction potential; P‐media, production/basal media; P/V, specific power input; *Q* ^2^, goodness of prediction; *R* ^2^, goodness of fit.

The P‐media quality indicators which are typically used in industrial media preparation such as osmolality and pH as well as the pCO_2_ concentration at the end of the media preparations were all affected by temperature, specific power input, and preparation time (Table 4). The single effects of the three factors were similar to each other for all three responses and a correlation analysis revealed that media osmolality (*R* ^2^:0.67) and media pH (*R* ^2^: 0.74) correlate with the pCO_2_ measurements in P‐media (data not shown). Lower pCO_2_ values at high temperature, high specific power input, and long preparation times derived from increased gas stripping effects over time as well as decreased solubilities of CO_2_ at these settings and therefore directly affected media pH and media osmolality. Different gas solubilities and gas transfer rates also apply for O_2_. Therefore, the average DO value during media preparation was influenced by temperature and power input as well. The DO value at the end of the media batch, however, seemed to be strongly affected by the oxidation reactions which might have plateaued at high temperature and long preparations times leading to an increase in DO at the end of these preparations as discussed earlier (Figure 3). This further explains the strong interaction term of temperature and preparation time in the data table for DO. In general, interaction effects could not be detected with the regularly applied media analytics, pH, and osmolality, but could be detected with ORP and 2‐DFL measurements. The ORP at the end of the P‐media batches varied strongly between −15 and −190 mV, depending on the applied preparation conditions. Therefore, different preparation parameter settings can lead to a more reducing or oxidizing environment resulting in different chemical properties of the media. Since the redox potential is influenced by pH and DO 30, it is affected by the same factors of the DoE (Table 4). The redox potential of the media before inoculation has been shown before to correlate with growth rates in animal cell culture 36 and lower culture redox potential led to higher specific growth rates and mAb concentrations in a hybridoma process 37. Whilst reducing conditions can have a positive effect on cell growth and product titer, it might further decrease mAb galactosylation 38. However, depending on media hold/storage‐times and conditions as well as media equilibration procedure, the impact of media preparation on the redox potential prior to inoculation might be negligible. LC‐MS measurements were conducted with the P‐media batches and the results were included in the DoE analysis. The data show chemical proof that depending on the preparation conditions, various media degradation processes can occur already during the media preparation process. l‐methionine sulfoxide is an oxidation product of methionine 33 and is therefore positively correlated with higher oxygen concentrations or specific power input. The negative effect of temperature on this variant could be due to a lower average DO or due to acceleration or preferation of other or further degradation pathways at these conditions. 5‐hydroxy‐l‐tryptophan can emerge via tryptophan oxidation 39, 40. The fact that all three factors P/V, preparation time, and temperature had a negative effect on the occurrence of this variant indicates that it is only an intermediate product and further reacts to N‐formylkynurenine as presented in the study by Rexroth et al. 39. In contrast to 5‐hydroxy‐l‐tryptophan, N‐formylkinurenine has been shown by McElearney et al. 41 to negatively affect cell growth in HEK‐293 cultures. The two vitamin B6 forms pyridoxal and pyridoxamine were affected by temperature and preparation time. Pyridoxal can readily undergo reactions with amino acids forming Schiff base and can subsequently further react to pyridoxamine 42, 43. The Schiff base reaction is known to be enhanced by heat and metal ions 42. However, no impact on process performance is expected since both forms, pyridoxal and pyridoxamine, are interconvertible in the cellular metabolism 42. The signal of the 2D FL probe was included into the DoE analysis with respect to the main peaks at ex450 nm/em530 nm and ex290 nm/em370 nm. Fluorescence signals are generally sensitive to temperature, pH, and concentration/presence of quenchers as molecular oxygen 44. Furthermore, degradation mechanisms of fluorescence substances such as tryptophan, tyrosine, and riboflavin 5, 9, 34 can occur during the preparation process, further affecting the fluorescence EEM. The fluorescence signal at em450 nm/ex530 nm was significantly affected by temperature, power input, preparation time and furthermore influenced by an interaction term of temperature and preparation time (Table 4). The fluorescence signal at ex290 nm/em370 nm was less sensitive to environmental changes and only affected by temperature. With the LC‐MS analysis it was possible to further detect significant changes in the media batches for taurine, a tyrosine variant, cyanocobalamin, riboflavin, and 3‐amino‐propionamide. By correlation of all analytes out of the LC‐MS measurements with PLS regression models to the 2D FL data, it was possible to gain deeper insight in the phenomenons that affect the media’ s fluorescence (Table 5). Methionine has a strong affinity to Cu^2+^ and therefore can reduce tryoptophane‐Cu(II) complexes which directly affects tryptophan fluorescence signals 45. Literature suggests that the same effects most probably apply for l‐methioninesulfoxide 46. Tyrosine is a fluorescent molecule 47 and therefore variants of this amino acids directly affect the fluorescent signals. Taurine can play an important role for antioxidant processes in cell cultivations 48. It is capable of building stable complexes with Cu^2+^ and therefore might affect tryptophan fluorescence in a similar way as described for methionine 49. Pyridoxamine, pyridoxal, riboflavin, and 5‐hydroxtryptophan are fluorescent compounds 47, 50, 51, 52 and therefore correlate strongly with the 2D FL spectra. 3‐Aminopropionamide is a product of the Maillard reaction. Maillard reaction products can undergo Cu^2+^ metal ion complexation 53 and thus might be correlated to fluoresence spectra. Cyanocobalamin can be readily degraded by light and under oxidizing conditions 42. Cobalamin has a strong absorbance at around 360 nm which can directly affect the emission at this wavelength from tryptophan and tyrosine 54. The results clearly demonstrate that 2D FL spectroscopy is a valuable tool to detect a wide variety of media changes. Furthermore, through the PLS regression with LC‐MS data quantitative predictions can be made for media batches.

**Table 5:** PLS regression model results for the P‐media out of the 2^nd^ study. 2D FL EEM at the end of the media preparation were correlated with LC‐MS data

Analyte	Range	*R* ^2^	*Q* ^2^	RMSEE	RMSEcv	Principal components
l‐methionine sulfoxide	44–100 [%]	0.98	0.83	2.40	7.50	3
Tyrosine variant	91–100 [%]	0.92	0.57	0.90	1.75	3
Taurine	86–100 [%]	0.84	0.56	1.56	2.37	2
Pyridoxamine	64–100 [%]	0.99	0.82	1.37	5.35	3
Pyridoxal	64–100 [%]	0.90	0.78	3.28	4.36	2
Cyanocobalamin	86–100 [%]	0.94	0.55	1.08	2.63	3
Riboflavin	83–100 [%]	0.99	0.81	0.60	2.98	4
5‐hydroxy‐l‐tryptophan	67–100 [%]	0.99	0.72	0.95	5.37	4
3‐amino‐propionamide	65–100 [%]	0.86	0.65	4.35	6.04	2

*Q* ^2^, goodness of prediction; *R* ^2^, goodness of fit; RMSEcv, root mean square error from cross‐validation; RMSEE, root mean square error of estimation.

Regarding the F‐media DoE evaluation, no effects could be detected with routine media quality indicators such as pH and osmolality (Table 6). Furthermore, the range of pCO_2_ measurements was much lower (1–4%) in F‐media when compared to the P‐media (7–32%), this is to some extent based on the different amount of sodium bicarbonate between F‐media and P‐media recipes. The data further assists the hypothesis that media osmolality and pH effects, in the P‐media DoE, mostly derived from variable pCO_2_ values. The tendency of the preparation parameters on DO and pCO_2_ concentrations were similar for P‐media and F‐media due to the general underlying physico‐chemical principles, e.g. higher temperature leads to lower gas solubilities. ORP and 2D FL signals could detect effects of the preparation parameters. However, the ranges of the measurements were lower than for P‐media. The single effects on ORP and 2D FL signals are partially contradicting to the P‐media results, due to the complexity of these responses and the fact that they are highly media recipe specific. No LC‐MS measurements were conducted for the F‐media batches.

**Table 6:** Impact of preparation parameters on physico‐chemical media characteristics (F‐media). The coefficients in terms of coded factors of the DoE are presented for F‐media. Confidence interval was set to 95% for all responses. Interaction effects are denoted as A × C

		(A)	(B)	(C)			
F‐media	Range	Temp. (30–37°C)	P/V (64–110 W/m^3^)	Prep. time (110–170 min.)	A × C	*R* ^2^	*Q* ^2^
Osmolality	1276–1341 [mOsm/kg]	–	–	–	–	–	–
pH	6.93–6.97 [‐]	–	–	–	–	–	–
pCO_2_	1–4 [%]	−0.74	−0.53	−0.69	–	0.93	0.88
ORP	−227–−253 [mV]	−9.44	0.84	−4.37	–	0.99	0.98
DO _average_	10–23 [%]	−2.2	3.51	–	–	0.89	0.85
2D‐FL (ex450/em530)	1215–1363 [IU]	30.78	–	50.14	12	0.95	0.92
2D‐FL (ex290/em370)	1242–1439 [IU]	−83.9	–	9.30	–	0.97	0.95

DO, dissolved oxygen; F‐media, feed media ORP, oxidation reduction potential; P‐media, production/basal media; P/V, specific power input; *Q* ^2^, goodness of prediction; *R* ^2^, goodness of fit.

#### 3.2.3 Impact on fed‐batch cell cultivations

**Figure 5:**
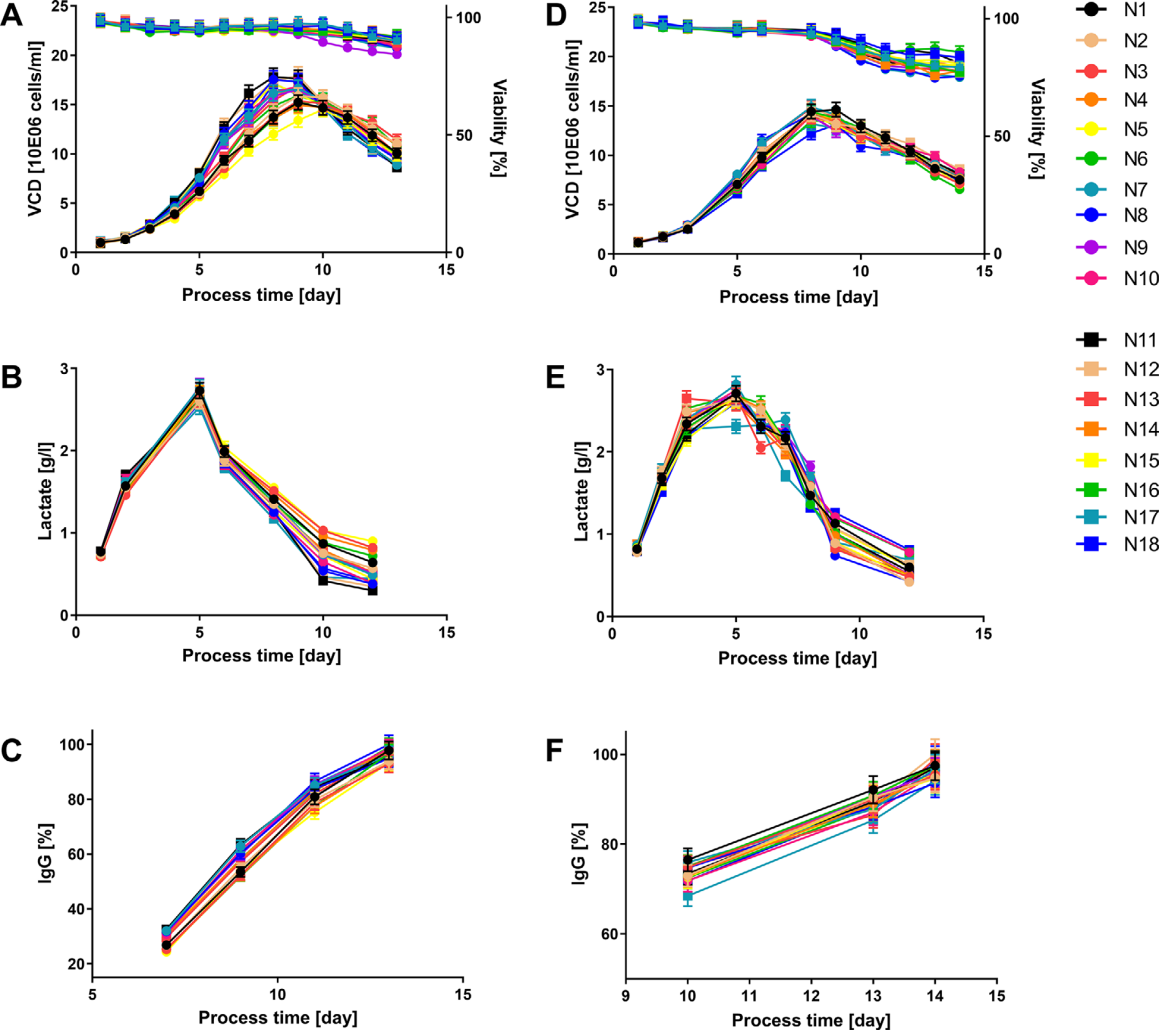
Fed‐batch process performance of cell line A with variable P‐media (A, B, C) and F‐media (D, E, F) out of the design of experiment. The legend corresponds to the media batch number out of Table 1. The error bars represent the technical measurement error of the analytical devices

Finally, the prepared media batches were used in fed‐batch cell cultivations in order to investigate the impact of media preparation variability on process performance. As can be seen in Figure 5A–C for P‐media and cell line A, certain variabilities in cell growth and peak VCD could be observed. Nonetheless, metabolic profiles and final product titer were similar between all cell culture processes and within each cell line. Therefore, the variability introduced during P‐media preparation did not strongly affect the overall process performance. The cultivations conducted with variable F‐media batches did show only slight variations in the viability profiles at the end of the process for cell line A but no differences in cell growth, metabolites, or product concentrations (Figure 5D–F). Finally, the F‐media batches, which were more robust to changes of the preparation parameters resulting in similar physico‐chemical characteristics (Figure 4), did show less variability in the final process performance when compared to the P‐media (Figures 3 and 5). Final product titer, harvest viability, and cell specific rates over the growth phase of the fed‐batch processes were included in the DoE analysis for P‐ and F‐media and for both cell lines. The significant models are presented in Table 7.

**Table 7:** Impact of preparation parameters and storage time on process performance. The coefficients in terms of coded factors of the DoE are presented for P‐ and F‐media. Confidence interval was set to 95% for all responses. Values in italic are not significant. Interaction effects are denoted as B × C

		(A)	(B)	(C)	(D)				
	Range	Temp. (28–38°C)	P/V (30–130 W/m^3^)	Prep. Time (65–160 min)	Storage time (4–18 days)	B × C	*D* ^2^	*R*²	*Q*²
**P‐media**									
q_lac_ (cell line B)	1.57–2.37 [pmol/cell/day]	–	–	–	0.29	–	–	0.60	0.48
Product titer (cell line A)	2.6–2.8 [g/l]	–	*0.016*	–	−0.086	–	–	0.59	0.40
µ_average_(cell line A)	0.29–0.41 [1/d]	–	−0.017	*0.096*	−0.034	−0.012	–	0.71	0.44
**F‐media**									
Harvest viability (cell line B)	79–88 [%]	*0.066*	−1.74	−1.57	*0.45*	–	3.26	0.78	0.40

F‐media, feed media; P‐media, production/basal media; P/V, specific power input; *Q* ^2^, goodness of prediction; *R* ^2^, goodness of fit.

Through the coded factors out of the DoE it could be revealed that the derived, cell line dependent, models for specific lactate production q_lac_ (cell line B), product titer (cell line A), and average specific cell growth µ_average_ (cell line A) were mostly affected by the storage time and not by the preparation parameters of the P‐media. Although strong effects on the chemical media composition could be observed already after the media preparation processes (Table 5) and the fact that several compounds have been reported before to have an impact on cell culture performance 40, 41, 48, potential effects of the media preparation parameters might have been masked by media storage or media equilibration conditions prior to inoculation. The impact of variable F‐media preparation parameters on process performance was negligible. The only model for cell line B that could be derived was for harvest cell viability which only varied between 79 and 88%. Final product quality assessment was performed for cell line A and fed‐batch processes N1, N3, N5, N7, N9, N11, N12, N13, and N17 for P‐media and batches N6, N8, N9, N11, N17, and N18 for F‐media. These processes showed the highest variability in physico‐chemical parameters at the end of the media preparation and/or in the corresponding fed‐batch processes. No apparent differences in mAb aggregation or fragmentation profile and charge or glycosylation variants could be detected within P‐ or F‐media cultivations (Supporting Information Table 2).

## 4 CONCLUDING REMARKS

Within this contribution, we compared two spectroscopic methods as online monitoring tools during cell culture media preparation. Hereby, MIR and 2D FL were applied online during the media preparation process. Variable media batches were prepared, through e.g. the usage of different basal powder suppliers. In contrast to the MIR analytics, the 2D FL spectroscopy showed superior sensitivity to the introduced variability, and data preprocessing steps were less complex. The online application of these spectroscopic tools not only allowed the differentiation of media batches at the end of the preparation process but the indication of the underlying root cause during the preparation process. In the second study of this publication, we investigated the effect of media preparation parameters that may vary during scale‐up such as temperature, preparation time, and power input on media quality. The DoE approach revealed strong effects of the preparation parameters on the physico‐chemical quality indicators as media osmolality, pH, ORP, DO, and 2D FL signals. Furthermore, with LC‐MS analytics strong differences in several media compounds and reaction products could be revealed. The data demonstrate that variable media preparation conditions as they might occur during scale‐up, can lead to variable physico‐chemical media characteristics. The observed effects were furthermore highly recipe/media specific. Advanced media preparation scale‐up strategies as well as modifications in media recipes (here P‐media) towards more robust media batches (here F‐media) might lead to increased media and process robustness. The impact of media preparation parameters on overall process performance and product quality was negligible for production and feed media for both tested cell lines. However, this is most probably media and recipe specific, as well as dependent on media hold‐times/storage conditions and media equilibration procedures prior to inoculation.

Finally, we could show that through the application of online spectroscopic tools, media variability could be detected already during the preparation process and that changes in several chemical components could be correlated with the 2D FL data. This methodology is in line with the PAT initiative 55 and can be extremely useful for troubleshooting activities and could act as a final release method for media batches. Additionally, the technology could serve as a characterization method for process transfer and scale‐up/scale‐down activities. The presented data further outlines the need for more advanced routine analytics and scale‐up strategies in cell culture media preparation processes.

## CONFLICT OF INTEREST

The authors have declared no conflict of interest.

## Supporting information


**Supplementary Table 1**. List of reported components included in the LC‐MS method.
**Supplementary Table 2**. PQ data of the conducted fed‐batch processes for cell line A.
