# Supplementary material for: Towards robust cell culture processes — Unraveling the impact of media preparation by spectroscopic online monitoring
Source: Eng Life Sci. 2019 Aug 22;19(10):666–80. doi: 10.1002/elsc.201900050 (PMC6999248; doi:10.1002/elsc.201900050)
Supplement: Supplementary file 1 — Supplementary Table 1. List of reported components included in the LC‐MS method. Supplementary Table 2. PQ data of the conducted fed‐batch processes for cell line A. [file ELSC-19-666-s001.pdf]

Supplementary Table 1. List of reported components included in the LC-MS method.

Analyte
L-methionine sulfoxide
Tyrosine variant
Taurine
Pyridoxamine
Pyridoxal
Cyanocobalamin
Riboflavin
5-hydroxyl- L-tryptophan
3-amino-propionamide

Supplementary Table 2. PQ data of the conducted fed-batch processes for cell line A.

	Aggregates/Fragments			Charge Variants				Glycosylation pattern							
**Sample ID**	**HMWC [%]**	**Monomer [%]**	**LMWC [%]**	**APG [%]**	**Main [%]**	**BPG [%]**	**Man 5 [%]**	**A2G 0 [%]**	**A2FG 0 [%]**	**A2G 1 [%]**	**A2FG 1 [%]**	**A2FG 1 [%]**	**A2G 2 [%]**	**A2FG 2 [%]**	**A2G 2 [%]**
N1_P-media	0.3	98.4	1.3	30.8	62.4	6.8	2.9	2.3	37.3	5.8	28.5	9.4	-	7.5	1.5
N3_P-media	0.4	98.4	1.3	30.7	62.8	6.5	3.0	2.2	36.6	5.8	28.7	9.7	-	7.4	-
N5_P-media	0.4	98.3	1.3	29.6	63.3	7	3.2	1.7	38.1	4.9	29.2	10.7	2.3	7.6	-
N7_P-media	0.4	98.3	1.4	34.4	59.8	5.8	3.6	1.9	37.3	5.0	28.8	10.0	-	7.7	0.8
N9_P-media	0.3	98.4	1.3	34.3	59.7	6	2.5	2.9	35.7	6.4	28.1	10.0	-	7.7	0.9
N11_P-media	0.4	98.2	1.4	35.5	58.6	5.9	4.3	1.6	37.4	5.1	28.8	10.0	1.9	7.5	-
N12_P-media	0.4	98.3	1.3	35.3	59	5.8	2.4	2.9	34.2	6.4	28.0	9.1	1.2	7.7	-
N13_P-media	0.4	98.3	1.3	33.3	60.8	5.9	2.6	2.3	35.8	5.9	29.6	10.6	2.1	7.9	-
N17_P-media	0.4	98.3	1.3	34.2	59.6	6.1	2.2	3.2	35.7	6.3	27.6	9.7	-	7.5	-
N6_F-media	0.5	98.1	1.4	37	57.4	5.6	4.1	3.8	36.8	4.6	26.0	9.5	1.2	7.1	-
N8_F-media	0.5	98.2	1.3	38	57	5.1	3.8	2.8	37.8	5.9	26.8	9.8	1.2	7.3	-
N9_F-media	0.5	98.2	1.3	36.7	58	5.4	2.5	3.4	37.2	8.5	25.9	9.6	-	6.1	0.8
N11_F-media	0.4	98.2	1.4	35.9	58.4	5.6	3.9	2.0	37.7	4.5	26.7	10.6	-	7.4	-
N17_F-media	0.3	98.2	1.4	32.7	60.8	6.5	3.0	1.8	36.9	6.5	28.0	10.9	1.6	7.4	-
N18_F-media	0.4	98.3	1.2	34.4	59.7	5.9	3.1	3.2	36.7	6.3	26.5	10.7	1.2	7.2	-
